# Adverse Health Outcomes Among US Testicular Cancer Survivors After Cisplatin-Based Chemotherapy vs Surgical Management

**DOI:** 10.1093/jncics/pkz079

**Published:** 2019-10-08

**Authors:** Vaibhav Agrawal, Paul C Dinh, Chunkit Fung, Patrick O Monahan, Sandra K Althouse, Kelli Norton, Clint Cary, Lawrence Einhorn, Sophie D Fossa, Nabil Adra, Lois B Travis

**Affiliations:** 1 Division of Hematology-Oncology, Department of Medicine, Indiana University School of Medicine, Indianapolis, IN; 2 Department of Biostatistics, Indiana University Melvin and Bren Simon Cancer Center, Indianapolis, IN; 3 Department of Epidemiology and Biostatistics, Indiana University of Public Health, Bloomington, IN; 4 Division of Hematology and Oncology, Department of Medicine, University of Rochester School of Medicine and Dentistry, James P. Wilmot Cancer Institute, Rochester, NY; 5 Department of Urology, Indiana University School of Medicine, Indianapolis, IN; 6 Department of Oncology, Oslo University Hospital, Radium Hospital, Oslo, Norway

## Abstract

We evaluated for the first time, to our knowledge, adverse health outcomes (AHOs) among US testicular cancer survivors (TCS) given chemotherapy (n = 381) vs surgery-only patients (n = 98) managed at a single institution, accounting for non-treatment-related risk factors to delineate chemotherapy’s impact. Chemotherapy consisted largely of bleomycin-etoposide-cisplatin (BEP) administered in three or four cycles (BEPx3, n = 235; BEPx4, n = 82). Incidence of at least 3 AHOs was lowest in surgery-only TCS and increased with BEPx3, BEPx4, and other cisplatin-based regimens (12.2%, 40.8%, 52.5%, 54.8%; *P *<* *.0001). Multivariable modeling assessed associations of risk factors and treatment with hearing impairment, tinnitus, peripheral neuropathy, and Raynaud phenomenon. Risk for each AHO statistically increased with both increasing chemotherapy burden (*P* < .0001) and selected modifiable risk factors (*P* < .05): hypertension (odds ratio [OR] = 2.40) and noise exposure (OR ≥ 2.3) for hearing impairment; noise exposure for tinnitus (OR ≥ 1.69); peripheral vascular disease for neuropathy (OR = 8.72); and current smoking for Raynaud phenomenon (OR = 2.41). Clinicians should manage modifiable risk factors for AHOs among TCS.

Testicular cancer is the most common cancer in men aged 18–39 years (1). Since cisplatin-based chemotherapy was introduced in the 1970s (2), the overall 5-year relative survival rate is over 95% (3). As a result, 1 in 600 US men is a testicular cancer survivor (TCS), representing a growing need to evaluate the subsequent development of adverse health outcomes (AHOs). Nonetheless, the few single-institution investigations of US TCS (4–8) have been limited in scope, generally either not addressing AHOs (5,7,8) or evaluating fewer than five conditions (6). Additionally, only Oh et al. (6) included a control group of TCS managed with surgical approaches alone but examined only four AHOs. 

In view of these gaps, our goal was to study AHOs among US TCS after contemporary cisplatin-based chemotherapy compared with a surgery-only cohort. The study was institutional review board-approved at Indiana University. Eligible TCS had a histologic or serological germ cell tumor diagnosis at 55 years or younger; all administered treatment and management was completed at least 1 year before enrollment. All participants were disease-free at routine follow-up and completed AHO-focused health questionnaires. AHO definitions and statistical methods are provided in the Supplementary Methods (available online). Two-sided *P* < .05 defined statistical significance.

Overall, 479 patients were evaluated (Table 1). Chemotherapy (n = 381) consisted largely of three or four cycles of bleomycin, etoposide, and cisplatin (BEPx3, n = 235; BEPx4, n = 82), with 64 patients receiving other cisplatin-based chemotherapy regimens (Other Plat). Median cumulative cisplatin doses were 300 mg/m^2^ (BEPx3) and 400 mg/m^2^ (BEPx4 and Other Plat). A total of 98 patients were managed only surgically. No patient received radiotherapy.

**Table 1.** Clinical and other characteristics and AHO in TCS by type of management

		Treatment regimen				
	All patients	Surgery	BEPx3	BEPx4	Other chemotherapy*	
Characteristic	n = 479	n = 98	n = 235	n = 82	n = 64	*P* †
Clinical characteristic						
Age at diagnosis, y						
Median [range]	31.0 [10.0, 56.2]	33.9 [15.4, 56.2]	30.0 [10.0, 53.0]	27.0 [16.0, 49.0]	31.5 [13.0, 55.0]	.0092
Age at clinical evaluation, y						
Median [range]	38.3 [18.3, 74.5]	39.0 [20.2, 62.3]	38.4 [18.3, 74.5]	36.3 [20.1, 71.3]	40.6 [20.8, 71.1]	.2468
Category						.3384
<20	3 (0.6%)	0	3 (1.3%)	0	0	
20–29	101 (21.1%)	20 (20.4%)	47 (20.0%)	21 (25.6%)	13 (20.3%)	
30–39	169 (35.3%)	35 (35.7%)	84 (35.7%)	32 (39.0%)	18 (28.1%)	
40–49	135 (28.2%)	30 (30.6%)	65 (27.7%)	23 (28.0%)	17 (26.6%)	
50+	71 (14.8%)	13 (13.3%)	36 (15.3%)	6 (7.3%)	16 (25.0%)	
Histologic type§						<.0001
Seminoma	127 (27.0%)	55 (56.7%)	52 (22.4%)	6 (7.4%)	14 (23.0%)	
Nonseminoma	344 (73.0%)	42 (43.3%)	180 (77.6%)	75 (92.6%)	47 (77.0%)	
Site‖						<.0001
Testis	397 (84.1%)	98 (100.0%)	205 (88.4%)	55 (67.9%)	39 (63.9%)	
Extragonadal	75 (15.9%)	0	27 (11.6%)	26 (32.1%)	22 (36.1%)	
Cisplatin-based chemotherapy						<.0001
BEP¶	329 (68.7%)	0	235 (100.0%)	82 (100.0%)	12 (18.8%)	
EP#	20 (4.2%)	0	0	0	20 (31.3%)	
None	98 (20.5%)	98 (100.0%)	0	0	0	
Other**	32 (6.7%)	0	0	0	32 (50.0%)	
Cumulative dose of cisplatin, mg/m^2^						
Median [range]	300 [0, 700]	0 [0, 0]	300 [276, 334]	400 [370, 414]	400 [198, 700]	<.0001
Category						<.0001
0	98 (20.5%)	98 (100.0%)	0	0	0	
<300	25 (5.2%)	0	16 (6.8%)	0	9 (14.1%)	
300	216 (45.1%)	0	212 (90.2%)	0	4 (6.3%)	
301–399	28 (5.8%)	0	7 (3.0%)	13 (15.9%)	8 (12.5%)	
** 400**	95 (19.8%)	0	0	62 (75.6%)	33 (51.6%)	
** >400**	17 (3.5%)	0	0	7 (8.5%)	10 (15.6%)	
Cumulative dose of bleomycin, IU						
Median [range]	270 000 [0.0, 630 000]	0.0 [0.0, 0.0]	270 000 [90 000, 270 000]	360 000 [0.0, 360 000]	0.0 [0.0, 630 000]	<.0001
Category						<.0001
0	143 (29.9%)	98 (100.0%)	0	2 (2.4%)	43 (67.2%)	
>0–180 000	27 (5.6%)	0	8 (3.4%)	8 (9.8%)	11 (17.2%)	
181 000–270 000	254 (53.0%)	0	227 (96.6%)	22 (26.8%)	5 (7.8%)	
271 000–360 000	52 (10.9%)	0	0	50 (61.0%)	2 (3.1%)	
360 000+	3 (0.6%)	0	0	0	3 (4.7%)	
Retroperitoneal lymph node dissection††						<.0001
No	303 (64.3%)	80 (81.6%)	157 (68.6%)	30 (37.0%)	36 (57.1%)	
Yes	168 (35.7%)	18 (18.4%)	72 (31.4%)	51 (63.0%)	27 (42.9%)	
Time from chemotherapy/surgery to clinical evaluation, y						
Median [range]	4.1 [1.0, 34.9]	3.8 [1.0, 30.7]	4.3 [1.0, 25.5]	4.5 [1.0, 34.9]	3.8 [1.0, 25.1]	.7518
Category						.0081
<2	149 (31.1%)	24 (24.5%)	79 (33.6%)	23 (28.0%)	23 (35.9%)	
2–5	136 (28.4%)	44 (44.9%)	55 (23.4%)	23 (28.0%)	14 (21.9%)	
6–9	70 (14.6%)	15 (15.3%)	36 (15.3%)	13 (15.9%)	6 (9.4%)	
≥10	124 (25.9%)	15 (15.3%)	65 (27.7%)	23 (28.0%)	21 (32.8%)	
Sociodemographic characteristic						
Race						.1817
White	453 (95.0%)	94 (95.9%)	225 (96.2%)	73 (90.1%)	61 (95.3%)	
Non-white	24 (5.0%)	4 (4.1%)	9 (3.8%)	8 (9.9%)	3 (4.7%)	
Marital status††						.4641
Not married	163 (34.2%)	32 (32.7%)	78 (33.3%)	34 (41.5%)	19 (30.2%)	
Married/living as married	314 (65.8%)	66 (67.3%)	156 (66.7%)	48 (58.5%)	44 (69.8%)	
Education§§						.2391
High school or less	85 (17.8%)	15 (15.3%)	35 (15.0%)	17 (20.7%)	18 (28.1%)	
Some college/college graduate	296 (62.1%)	62 (63.3%)	146 (62.7%)	51 (62.2%)	37 (57.8%)	
Post-graduate level/other	96 (20.1%)	21 (21.4%)	52 (22.3%)	14 (17.1%)	9 (14.1%)	
Noise exposure‖‖						.0073
None	236 (49.9%)	60 (61.2%)	119 (51.1%)	30 (37.5%)	27 (43.5%)	
Work-related only	111 (23.5%)	22 (22.4%)	60 (25.8%)	17 (21.3%)	12 (19.4%)	
Non-work related only	41 (8.7%)	3 (3.1%)	21 (9.0%)	10 (12.5%)	7 (11.3%)	
Both	85 (18.0%)	13 (13.3%)	33 (14.2%)	23 (28.8%)	16 (25.8%)	
Physical examination results						
Body mass index, kg/m^2^¶¶						
Median [range]	28.3 [18.0, 66.6]	28.0 [18.0, 54.3]	28.2 [19.0, 66.6]	28.2 [20.5, 46.1]	29.2 [20.0, 42.0]	.6641
Category						.5166
<25 (normal)	105 (22.2%)	24 (25.3%)	54 (23.2%)	16 (19.8%)	11 (17.2%)	
25≤30 (overweight)	194 (41.0%)	40 (42.1%)	95 (40.8%)	33 (40.7%)	26 (40.6%)	
30≤40 (obese)	148 (31.3%)	28 (29.5%)	66 (28.3%)	29 (35.8%)	25 (39.1%)	
≥40 (morbidly obese)	26 (5.5%)	3 (3.2%)	18 (7.7%)	3 (3.7%)	2 (3.1%)	
Health behavior						
Smoking status##						.0938
Never smoker	284 (59.4%)	60 (61.2%)	150 (64.1%)	45 (54.9%)	29 (45.3%)	
Former smoker	147 (30.8%)	31 (31.6%)	59 (25.2%)	30 (36.6%)	27 (42.2%)	
Current smoker	47 (9.8%)	7 (7.1%)	25 (10.7%)	7 (8.5%)	8 (12.5%)	
Average number of alcoholic drinks in past year***						.3286
Rarely/never	147 (30.8%)	30 (30.6%)	68 (29.1%)	29 (35.4%)	20 (31.3%)	
≤4/wk	182 (38.1%)	46 (46.9%)	90 (38.5%)	23 (28.0%)	23 (35.9%)	
5/wk–1/d	92 (19.2%)	16 (16.3%)	44 (18.8%)	20 (24.4%)	12 (18.8%)	
≥2/d	57 (11.9%)	6 (6.1%)	32 (13.7%)	10 (12.2%)	9 (14.1%)	
Moderate-intensity exercise†††						.0759
Yes	450 (94.5%)	95 (97.9%)	221 (94.0%)	79 (96.3%)	55 (88.7%)	
No	26 (5.5%)	2 (2.1%)	14 (6.0%)	3 (3.7%)	7 (11.3%)	
Vigorous-intensity exercise‡‡‡						.0664
Yes	313 (65.8%)	69 (71.1%)	159 (67.7%)	53 (64.6%)	32 (51.6%)	
No	163 (34.2%)	28 (28.9%)	76 (32.3%)	29 (35.4%)	30 (48.4%)	
AHO						
Total number of AHO						
Median [range]	2.0 [0.0, 11.0]	1.0 [0.0, 11.0]	2.0 [0.0, 10.0]	3.0 [0.0, 9.0]	3.0 [0.0, 9.0]	<.0001
Category						<.0001
0	82 (17.1%)	33 (33.7%)	41 (17.4%)	5 (6.1%)	3 (4.7%)	
1	115 (24.0%)	39 (39.8%)	47 (20.0%)	20 (24.4%)	9 (14.1%)	
2	96 (20.0%)	14 (14.3%)	51 (21.7%)	14 (17.1%)	17 (26.6%)	
3	66 (13.8%)	7 (7.1%)	32 (13.6%)	15 (18.3%)	12 (18.8%)	
4	43 (9.0%)	2 (2.0%)	25 (10.6%)	10 (12.2%)	6 (9.4%)	
5 or more	77 (16.1%)	3 (3.1%)	39 (16.6%)	18 (22.0%)	17 (26.6%)	
Type of AHO						
Tinnitus§§§						<.0001
Yes	176 (36.8%)	16 (16.3%)	92 (39.3%)	36 (43.9%)	32 (50.0%)	
No	302 (63.2%)	82 (83.7%)	142 (60.7%)	46 (56.1%)	32 (50.0%)	
Hearing impairment‖‖‖						<.0001
Yes	152 (33.2%)	11 (11.5%)	80 (36.0%)	33 (41.8%)	28 (45.9%)	
No	306 (66.8%)	85 (88.5%)	142 (64.0%)	46 (58.2%)	33 (54.1%)	
Peripheral neuropathy¶¶¶						<.0001
Yes	126 (26.5%)	4 (4.1%)	65 (27.8%)	27 (33.8%)	30 (46.9%)	
No	349 (73.5%)	93 (95.9%)	169 (72.2%)	53 (66.3%)	34 (53.1%)	
Ototoxicity and peripheral neuropathy						<.0001
Yes	80 (16.7%)	1 (1.0%)	41 (17.4%)	16 (19.5%)	22 (34.4%)	
No	399 (83.3%)	97 (99.0%)	194 (82.6%)	66 (80.5%)	42 (65.6%)	
Raynaud phenomenon###						<.0001
Yes	86 (18.2%)	2 (2.0%)	47 (20.3%)	24 (29.6%)	13 (20.6%)	
No	387 (81.8%)	96 (98.0%)	184 (79.7%)	57 (70.4%)	50 (79.4%)	
Hypogonadism with testosterone therapy****						.3004
Yes	52 (10.9%)	8 (8.2%)	28 (12.0%)	6 (7.4%)	10 (15.9%)	
No	423 (89.1%)	90 (91.8%)	205 (88.0%)	75 (92.6%)	53 (84.1%)	
Erectile dysfunction††††						.0548
Yes	67 (14.0%)	8 (8.2%)	30 (12.8%)	15 (18.5%)	14 (21.9%)	
No	410 (86.0%)	89 (91.8%)	205 (87.2%)	66 (81.5%)	50 (78.1%)	
Hypertension and on prescription medication‡‡‡‡						.0007
Yes	57 (12.2%)	0	36 (15.7%)	12 (15.0%)	9 (15.0%)	
No	410 (87.8%)	97 (100.0%)	194 (84.3%)	68 (85.0%)	51 (85.0%)	
Hypercholesterolemia and on prescription medication§§§§						.7902
Yes	58 (12.2%)	14 (14.3%)	25 (10.8%)	10 (12.2%)	9 (14.1%)	
No	418 (87.8%)	84 (85.7%)	207 (89.2%)	72 (87.8%)	55 (85.9%)	
CVD ‖‖‖‖						.0648
Yes	27 (5.7%)	1 (1.0%)	13 (5.6%)	8 (9.9%)	5 (7.9%)	
No	448 (94.3%)	97 (99.0%)	220 (94.4%)	73 (90.1%)	58 (92.1%)	
Peripheral vascular disease¶¶¶¶						.1063
Yes	19 (4.0%)	1 (1.0%)	8 (3.4%)	5 (6.2%)	5 (8.1%)	
No	455 (96.0%)	97 (99.0%)	225 (96.6%)	76 (93.8%)	57 (91.9%)	
Thromboembolic disease####						
Yes	0	0	0	0	0	
No	474 (100.0%)	98 (100.0%)	233 (100.0%)	81 (100.0%)	62 (100.0%)	
Renal disease*****						.1983
Yes	6 (1.3%)	0	4 (1.7%)	0	2 (3.3%)	
No	462 (98.7%)	97 (100.0%)	227 (98.3%)	80 (100.0%)	58 (96.7%)	
Diabetes and on prescription medication†††††						.4671
Yes	14 (3.0%)	5 (5.1%)	6 (2.6%)	1 (1.2%)	2 (3.2%)	
No	460 (97.0%)	93 (94.9%)	227 (97.4%)	80 (98.8%)	60 (96.8%)	
Thyroid disease†††††						.6403
Yes	5 (1.1%)	2 (2.0%)	2 (0.9%)	1 (1.2%)	0	
No	469 (98.9%)	96 (98.0%)	231 (99.1%)	80 (98.8%)	62 (100.0%)	
Problems with balance/vertigo/dizziness‡‡‡‡‡						.0483
Yes	55 (11.8%)	6 (6.1%)	25 (11.0%)	15 (19.5%)	9 (14.3%)	
No	410 (88.2%)	92 (93.9%)	202 (89.0%)	62 (80.5%)	54 (85.7%)	
Psychotropic prescription medication for anxiety and/or depression§§§§§						.1420
Yes	58 (12.1%)	9 (9.2%)	25 (10.6%)	11 (13.4%)	13 (20.3%)	
No	421 (87.9%)	89 (90.8%)	210 (89.4%)	71 (86.6%)	51 (79.7%)	

* Patients in this category received BEP chemotherapy other than three or four cycles (n = 12), EP chemotherapy (n = 20), and other cisplatin-based regimens (n = 32). AHO = adverse health outcome; BEPx3 = three cycles of bleomycin, etoposide, and cisplatin; BEPx4 = four cycles of bleomycin, etoposide, and cisplatin; CVD = cardiovascular disease; EPx4 = four cycles of etoposide and cisplatin; TCS = testicular cancer survivors; VeIP = vinblastine, ifosfamide, cisplatin; VIP = etoposide, cisplatin, ifosfamide.

† *P* value comparisons are based on χ^2^ test for categorical variables and Kruskal-Wallis test (normal approximation) for continuous variables. Missing values were not used in the calculation of the *P* values.

§ Includes eight participants with not-otherwise-specified germ cell tumor or unknown histology.

‖ Includes seven patients with primary site not stated.

¶ The standard BEP chemotherapy cycle that all our patients received consists of bleomycin 30 000 IU days 1, 8, 15; etoposide 100 mg/m^2^ days 1 through 5; and cisplatin 20 mg/m^2^ days 1 through 5. Includes 12 patients who received BEP other than three or four cycles.

# The standard dosing and standard EP schedule that all of our patients consists of etoposide 100 mg/m^2^ days 1 through 5 and cisplatin 20 mg/m^2^ days 1 through 5.

** Includes 32 patients with other cisplatin-based regimens: 11 participants with VIP and 1 with VeIP. Each standard VIP chemotherapy cycle that our patients received consists of etoposide 75 mg/m^2^ days 1 through 5, cisplatin 100 mg/m^2^ days 1through 5, and ifosfamide 1200 mg/m^2^ days 1 through 5.

†† Includes eight patients for whom retroperitoneal lymph node dissection status was not available.

‡‡ Includes two participants with marital status not available.

§§ Includes two patients for whom education level was not available.

‖‖ Noise exposure data were not available for six patients.

¶¶ Includes six patients with body mass index information not available.

## Reported health behavior was not available for one patient.

*** Reported health behavior was not available for one patient.

††† Reported health behavior was not available for three patients.

‡‡‡ Reported health behavior was not available for three patients.

§§§ Category includes one patient for whom this outcome was not available.

‖‖‖ Category includes 21 patients for whom this outcome was not available.

¶¶¶ Category includes four patients for whom this outcome was not available.

### Category includes six patients for whom this outcome was not available.

**** Category includes four patients who received bilateral orchiectomy that were not included in the comparison of hypogonadism, but were included elsewhere if the data was available.

†††† Category includes two patients for whom this outcome was not available.

‡‡‡‡ Category includes 12 patients for whom this outcome was not available.

§§§§ Category includes three patients for whom this outcome was not available.

‖‖‖‖ Category includes four patients for whom this outcome was not available. Includes coronary artery disease, heart failure, and cerebrovascular disease (categories not mutually exclusive and each category was counted as one AHO).

¶¶¶¶ Category includes five patients for whom this outcome was not available.

#### Category includes five patients for whom this outcome was not available.

***** Category includes 11 patients for whom this outcome was not available.

††††† Category includes five patients for whom this outcome was not available.

‡‡‡‡‡ Category includes five patients for whom this outcome was not available.

§§§§§ Category includes 14 patients for whom this outcome was not available.

‖‖‖‖‖ Participants could report more than one psychotropic medication.

Median age at evaluation (overall = 38.3 years) and median time since chemotherapy or surgery completion (overall = 4.1 years) were similar between groups. Surgery-only patients had a smaller percentage of nonseminomatous histology compared with chemotherapy-treated TCS receiving BEPx3, BEPx4, and Other Plat (43.3% vs 77.6%, 92.6%, 77.0%, respectively; *P* < .0001) and were more likely to have primary testicular germ cell tumor (100% vs 88.4%, 67.9%, 63.9%, respectively; *P* < .0001). Other clinical characteristics, physical examination results, and health behaviors were similar between groups, but more surgery-only TCS reported the absence of noise exposure than did chemotherapy-treated TCS (61.2% vs 51.1%, 37.5%, 43.5%, respectively; *P* = .0073). The median number of AHOs increased from 1 (range = 0–11) after surgery-only to 2 (range = 0–10) after BEPx3 and 3 (range = 0–9) after BEPx4 and Other Plat. Fewer TCS had at least 3 AHOs after surgery-only (12.2%) than after BEPx3 (40.8%), BEPx4 (52.5%), and Other Plat (54.8%) (*P* < .0001). Significant differences between surgery-only, BEPx3, BEPx4, and Other Plat were observed for the following AHOs (each *P* < .05): hearing impairment (11.5%, 36.0%, 41.8%, 45.9%); tinnitus (16.3%, 39.3%, 43.9%, 50.0%); peripheral neuropathy (4.1%, 27.8%, 33.8%, 46.9%); hypertension (0%, 15.7%, 15.0%, 15.0%); Raynaud phenomenon (2.0%, 20.3%, 29.6%, 20.6%); and balance/vertigo/dizziness (6.1%, 11.0%, 19.5%, 14.3%). For cardiovascular disease (CVD), differences were of borderline significance (1.0%, 5.6%, 9.9%, 7.9%; *P* = .0648).

Table 2 shows the results of multivariable modeling for selected AHOs. TCS given BEPx3, BEPx4, and Other Plat experienced significant tinnitus excesses (odds ratio [OR] = 3.0, 3.71, and 3.99 [*P* = .0005 each]), with risk increased by 1.44-fold (*P* < .0001) for each 100 mg/m^2^ of cisplatin. Prior work-related (OR = 1.69, *P* = .0426), non-work-related (OR = 2.1, *P* = .0399), and cumulative (OR = 2.13, *P* = .0078) noise exposures were associated with statistically increased twofold tinnitus risks, but no interaction with cisplatin dose existed (*P* = .5892).

**Table 2.** Logistic multivariable regression analyses of selected AHO in 479 TCS

Characteristic	AHO							
	Tinnitus: yes∗		Hearing loss: yes∗		Raynaud phenomenon: yes∗		Peripheral neuropathy: yes∗	
	OR (95% CI)	*P*	OR (95% CI)	*P*	OR (95% CI)	*P*	OR (95% CI)	*P*
Clinical characteristic								
Treatment		*P*_overall_ = .0011		*P*_overall_ = .0016		*P*_overall_ = .0014		*P*_overall_ <.0001
Surgery only	Ref.		Ref.		Ref.		Ref.	
BEPx3†	3.00 (1.61 to 5.60)	.0005	3.52 (1.71 to 7.28)	.0007	11.85 (2.74 to 51.29)	.0009	8.93 (3.04 to 26.29)	<.0001
BEPx4	3.71 (1.77 to 7.78)	.0005	3.80 (1.63 to 8.85)	.0020	20.56 (4.49 to 94.19)	<.0001	12.82 (4.01 to 40.94)	<.0001
Other chemotherapy‡	3.99 (1.83 to 8.72)	.0005	5.20 (2.14 to 12.63)	.0003	12.04 (2.52 to 57.62)	.0018	17.63 (5.47 to 56.83)	<.0001
Age at clinical evaluation, per 5 y	1.11 (0.99 to 1.26)	.0764	1.16 (1.02 to 1.33)	.0259	1.02 (0.87 to 1.18)	.8260	1.23 (1.07 to 1.41)	.0033
Time since chemotherapy completion, per 1 y	1.00 (0.96 to 1.04)	.9636	1.01 (0.97 to 1.06)	.4898	1.02 (0.97 to 1.06)	.5031	0.96 (0.92 to 1)	.0658
Cumulative dose of cisplatin, per 100 mg/m^2^§	1.44 (1.22 to 1.69)	<.0001	1.40 (1.17 to 1.66)	.0002	§	§	1.75 (1.41 to 2.17)	<.0001
Health behavior								
Smoking status		P_overall_ = .4704		*P*_overall_ = .6835		*P*_overall_ = .0402		*P*_overall_ = .5185
Never smoker	Ref		Ref		Ref		Ref	
Former smoker	0.83 (0.52 to 1.31)	.4261	0.81 (0.49 to 1.33)	.4002	0.93 (0.52 to 1.67)	.8146	1.27 (0.75 to 2.12)	.3740
Current smoker	1.27 (0.65 to 2.48)	.4800	0.85 (0.40 to 1.78)	.6655	2.41 (1.16 to 5.02)	.0183	1.43 (0.68 to 2.99)	.3485
Average no. alcoholic drinks in past year	‖	‖	‖	‖	‖	‖		*P*_overall_ = .6080
Rarely or never	‖	‖	‖	‖	‖	‖	Ref	
≤4/wk	‖	‖	‖	‖	‖	‖	1.08 (0.6, 1.94)	.7885
5/wk–1/d	‖	‖	‖	‖	‖	‖	1.25 (0.63, 2.48)	.5178
≥2/d	‖	‖	‖	‖	‖	‖	1.61 (0.78, 3.34)	.2017
AHO specific risk factor								
Cumulative dose of bleomycin, per 90 000 IU§	‖	‖	‖	‖	1.36 (1.12 to 1.65)	.0016	‖	‖
Noise exposure		*P*_overall_ = .0189		*P*_overall_ = .0003				
None	Ref		Ref		‖	‖	‖	‖
Work-related noise only	1.69 (1.02 to 2.79)	.0426	2.30 (1.32 to 4.00)	.0033	‖	‖	‖	‖
Non-work-related noise only	2.1 (1.03 to 4.25)	.0399	3.64 (1.70 to 7.81)	.0009	‖	‖	‖	‖
Both	2.13 (1.22 to 3.72)	.0078	2.75 (1.49 to 5.06)	.0012	‖	‖	‖	‖
Hypertension	1.08 (0.57 to 2.02)	.8175	2.40 (1.23 to 4.67)	.0101	1.13 (0.53 to 2.39)	.7529	1.23 (0.62 to 2.43)	.5468
CVD	1.33 (0.57 to 3.13)	.5118	1.05 (0.41 to 2.68)	.9245	0.58 (0.18 to 1.91)	.3720	0.95 (0.36 to 2.48)	.9086
Peripheral vascular disease	‖	‖	‖	‖	0.84 (0.22 to 3.12)	.7895	8.72 (2.41 to 31.62)	.0010
Diabetes and on prescription medication	‖	‖	‖	‖	2.23 (0.47 to 10.66)	.3164	1.53 (0.37 to 6.35)	.5614

* Ref. = “no.” AHO = adverse health outcome; BEP = bleomycin, etoposide, cisplatin; CI = confidence interval; CVD = cardiovascular disease; EP = etoposide, cisplatin; IU = international unit; OR = odds ratio; TCS = testicular cancer survivors; VeIP = vinblastine, ifosfamide, cisplatin; VIP = etoposide, cisplatin, ifosfamide.

† The standard BEP chemotherapy cycle that all of our patients received consists of bleomycin 30 000 IU days 1, 8, 15; etoposide 100 mg/m^2^ days 1 through 5; and cisplatin 20 mg/m^2^ days 1 through 5.

‡ Includes 32 patients with other cisplatin-based regimens: 11 participants with VIP and one with VeIP. Each standard VIP chemotherapy cycle that our patients received consists of etoposide 75 mg/m^2^ days 1 through 5, cisplatin 100 mg/m^2^ days 1through 5, and ifosfamide 1200 mg/m^2^ days 1 through 5.

§ Reported results are calculated utilizing the statistical model that included the cumulative dose variable instead of the treatment group variable. Please refer to Supplementary Methods (available online) for details on the statistical analysis modeling used.

‖ Variable not included in the analysis for the indicated AHO. Please refer to Supplementary Methods (available online).

Hearing loss increased statistically with increasing age at clinical evaluation (OR = 1.16 per 5 years, *P* = .0259), hypertension (OR = 2.40, *P* = .0101), and in each chemotherapy group (*P* < .01 each). With each 100-mg/m^2^ increase in cisplatin, hearing loss increased by 1.4-fold (*P* = .0002). Work-related (OR = 2.30, *P* = .0033), non-work-related (OR = 3.64, *P* = .0009), and cumulative (OR = 2.75, *P* = .0012) noise exposures conferred increased hearing loss, but no interaction with cisplatin dose existed (*P* = .3672).

Raynaud phenomenon was increased 12-fold (*P* = .0009) and 21-fold (*P* < .0001) following BEPx3 and BEPx4, respectively, and 12-fold after Other Plat (*P* = .0018). Risk increased with increasing bleomycin dose (OR = 1.36 per 90 000 IU, *P* = .0016). Current smoking was associated with statistically increased 2.4-fold risks.

Peripheral neuropathy was increased by 9-, 13-, and 18-fold, respectively, after BEPx3, BEPx4, and Other Plat (*P* < .0001 each). Increasing age at assessment (OR = 1.23 per 5 years, *P* = .0033), higher cumulative cisplatin dose (OR = 1.75 per 100 mg/m^2^, *P* < .0001), and peripheral vascular disease (OR = 8.72, *P* = .0010) were associated with higher neuropathy risks.

Our study investigates for the first time, to our knowledge, the prevalence and risk factors for chemotherapy-related AHOs in US TCS with surgery-only patients as the control group. We took into account previously identified AHO-specific risk factors, thus furthering our understanding of the specific impact of chemotherapy. Risk of treatment-related AHOs increased proportionately with increasing chemotherapy burden. Importantly, modifiable risk factors were also associated with AHOs even when controlled for chemotherapy.

It is noteworthy that even after a short median follow-up, about 1 in 7 TCS in each chemotherapy group had hypertension compared with no cases after surgery-only (*P* = .0007). A similar trend of borderline significance was noted for CVD.

Major strengths of our study are that all TCS were managed at one large, well-established US institution. However, AHOs were largely self-reported without baseline data pretherapy. Cross-sectional designs have potential inherent limitations and do not permit causal inference, although prospective follow-up is planned. Although the control group (surgery-only patients) had a lower disease burden initially than did chemotherapy-treated TCS, no TCS had active disease at the time of study enrollment. We cannot rule out, however, any potential influence of initial disease burden on AHO development.

Nonetheless, we provide for the first time, to our knowledge, estimates of the magnitude of AHOs associated with current chemotherapy in US TCS compared with surgery-only patients. This may potentially guide clinical decision-making, such as recommending surgical approaches with primary retroperitoneal lymph node dissection in patients with low-bulk stage II disease in an attempt to avoid chemotherapy-related AHOs. Future studies should address AHO incidence when adjuvant chemotherapy is applied in early-stage disease or in guiding decisions regarding primary retroperitoneal lymph node dissection vs chemotherapy in early stage II disease.

The incidence of several AHOs (eg, hypertension, CVD) will likely increase with the aging process (9). Thus, patients should be encouraged to adopt practices consistent with a healthy lifestyle and avoid noise exposure, ototoxic drugs, and other factors that may further affect AHOs. Health-care providers should diligently manage comorbidities to minimize the development or exacerbation of AHOs.

## Funding

This work was supported by the National Cancer Institute (1R01 CA157823 to LBT) and funds provided by Indiana University to NA.

## Notes

The authors of this study do not have any conflicts of interest to disclose in the subject matter or materials discussed in this article.

Our results were presented in part at the American Society of Clinical Oncology Annual Meeting, June 2018.

## Supplementary Material

pkz079_Supplementary_Data
