# Supplementary material for: Adverse Health Outcomes Among US Testicular Cancer Survivors After Cisplatin-Based Chemotherapy vs Surgical Management
Source: JNCI Cancer Spectr. 2019 Oct 8;4(2):pkz079. doi: 10.1093/jncics/pkz079 (PMC7065712; doi:10.1093/jncics/pkz079)
Supplement: pkz079_Supplementary_Data [file pkz079_supplementary_data.docx]

**SUPPLEMENTARY METHODS:**

Of patients who met eligibility criteria for this study, the participation rate for the chemotherapy-treated and surgery-only patients was 99.0% (381 enrolled of 385 approached) and 99.0% (98 enrolled of 99 approached), respectively. All participants provided informed consent. Abstracted data from medical records included information regarding GCT diagnosis and management, including cumulative dose of all cytotoxic drugs.

Categorical and continuous/ordinal data were described using counts (percentages) and medians (ranges), respectively. Chemotherapy-treated patients in this report were previously included in the multi-institutional study by Fung et al. (10), and AHOs were defined as in this prior investigation (10). Sociodemographic, health behavior, treatment variables and AHOs were tested for associations with treatment groups using Kruskal-Wallis, Pearson’s chi-square or Fisher’s exact test as appropriate in Table 1 (11). Multivariable logistic regression models assessed associations with treatment groups and selected risk factors as independent variables and specific AHOs as dependent variables.

In Table 2, AHO (tinnitus, hearing loss, Raynaud phenomenon, and peripheral neuropathy) were selected for additional analyses based on results of prior reports. Cisplatin has treatment-related toxicities of hearing loss (12), tinnitus (12), and peripheral neuropathy (13); while bleomycin is associated with Raynaud phenomenon (14). Non-treatment AHO-specific risk factors (including smoking status, alcohol use, noise exposure, hypertension, cardiovascular disease, peripheral vascular disease, and diabetes) were chosen a priori for each AHO, based on a literature review of risk factors associated with de novo forms of the conditions. Tinnitus and hearing loss have previously been associated with smoking (15), noise exposure (16,17), hypertension (18), and cardiovascular disease (19). Raynaud phenomenon has been reported to be associated with smoking (20,21) and hypertension (22), and is a complication that has been shown to be more prevalent in patients with peripheral vascular disease (20) and diabetes (23). Peripheral neuropathy has previously been associated with smoking (24), alcohol use (24), hypertension (24), cardiovascular disease (25), and peripheral vascular disease (26), and diabetes (27,28).

In Table 2, due to collinearity with treatment group, separate multivariable logistic regression models assessed associations with cumulative cisplatin dose (or cumulative bleomycin dose for Raynaud phenomenon) for AHOs. Possible interactions between cumulative cisplatin dose and noise exposure were tested with two-way interaction terms in models for tinnitus and hearing loss. An alpha of 0.05 was used for all tests. All tests were two-sided. All analyses were performed in SAS 9.4 (29).
